# Trends in maintenance status and usability of public automated external defibrillators during a 5-year on-site inspection

**DOI:** 10.1038/s41598-022-14611-1

**Published:** 2022-06-24

**Authors:** Tae Youn Kim, Yun-kyoung Jung, Sun Hwa Yoon, Sun Ju Kim, Kyoung-chul Cha, Woo Jin Jung, Young Il Roh, Soyeong Kim, Sung Hwa Kim, Dae Ryong Kang, Sung Oh Hwang

**Affiliations:** 1 Department of Emergency Medicine, Dongguk University College of Medicine, Dongguk University Ilsan Hospital, Goyang, South Korea; 2 Korean Association for Safe Communities, Seoul, South Korea; 3 Department of Emergency Medicine, Yonsei University Wonju College of Medicine, 20 Ilsanro, Wonju, 26427 South Korea; 4 Department of Biostatistics, Yonsei University Wonju College of Medicine, Wonju, Republic of Korea

**Keywords:** Cardiology, Public health

## Abstract

This study aimed to assess the trend of the maintenance status and usability of public automated external defibrillators (AEDs). Public AEDs installed in Seoul from 2013 to 2017 were included. An inspector checked the maintenance status and usability of the AEDs annually using a checklist. During the study period, 23,619 AEDs were inspected. Access to the AEDs was improved, including the absence of obstacles near the AEDs (from 90.2% in 2013 to 99.1% in 2017, p < 0.0001) and increased AED signs (from 34.3% in 2013 to 91.3% in 2017, p < 0.0001). The rate of AEDs in normal operation (from 94.0% in 2013 to 97.6% in 2017, p < 0.0001), good battery status (from 95.6% in 2013 to 96.8% in 2017, p = 0.0016), and electrode availability increased (from 97.1% in 2013 to 99.0% in 2017, p < 0.0001); the rate of electrode validity decreased (from 90.0% in 2013 to 87.2% in 2017, p < 0.0001). The overall rate of the non-ready-to-use AEDs and AEDs with less than 24-h usability accounted for 15.4% and 44.1% of the total number of AEDs, respectively. Although most AEDs had a relatively good maintenance status, a significant proportion of public AEDs were not available for 24-h use. Invalid electrodes and less than 24-h accessibility were the main reasons that limited the 24-h usability of public AEDs.

## Introduction

Immediate cardiopulmonary resuscitation (CPR) and rapid defibrillation are the most important measures to save patients with cardiac arrest from ventricular fibrillation^1^. Placement of automated external defibrillators (AEDs) in public places is an important way of providing rapid defibrillation to patients with out-of-hospital cardiac arrest (OHCA). By enabling the general public to perform defibrillation, the survival rate of such patients can be increased^2–5^. It has been reported that the survival rate of patients with OHCA improved in a community with public access defibrillation (PAD) programs where AEDs were installed in public places to increase the chances of survival^6–8^. Accordingly, the number of AEDs being installed in public places is increasing with the spread of the PAD programs. However, AED devices might be prone to failure or malfunction^9,10^.

### Importance

Good maintenance of AEDs is important to ensure reliable operation and reduce the risk of failure or malfunctioning of the AEDs. The defibrillators in medical facilities or ambulances are used frequently and regularly undergo maintenance checks performed by experts. Contrarily, public AEDs may not be maintained efficiently since they are not used frequently or checked by experts. Not only do the AEDs need to be well maintained to avoid failure or malfunction, but their accessibility should be enhanced to increase their usability.

With the increase in the number of publicly installed AEDs, communities need to pay attention to the maintenance of AEDs installed in their jurisdictions to ensure that they are always available and ready for use.

### Goal of this investigation

In this context, it is necessary to assess the maintenance status and availability of AEDs in public places and determine strategies for good maintenance in the process of disseminating PAD programs. However, little information is available regarding the maintenance and availability of AEDs installed in public places. Thus, the objective of this study was to investigate the trends of the management status and usability of AEDs installed in public places and provide basic data for establishing future management plans for AEDs.

## Results

**Figure 1:**
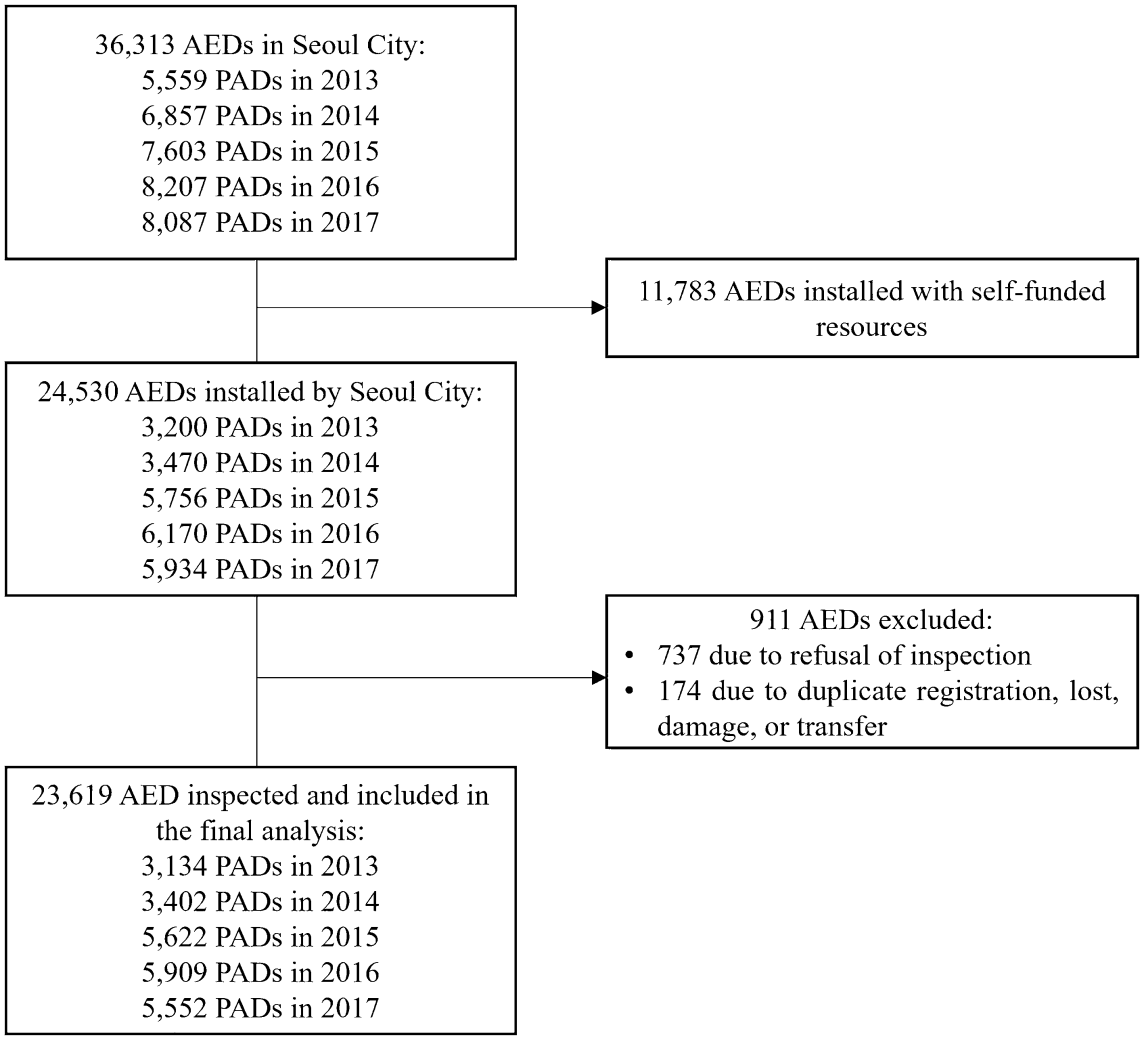
Flow chart showing the enrollment process for AEDs. AED, automated external defibrillator.

Among the 36,313 AEDs, which included both the AEDs already on-site and those newly installed during the study period, 11,783 (32.4%) AEDs installed with self-funded resources were excluded. A total of 911 (2.5%) AEDs that could not be inspected were also excluded (737 for refusal of inspection; 174 for duplicate data, lost data, demolition, or transfer). Finally, 23,619 AEDs, including 3134 (13.3%) in 2013, 3402 (14.4%) in 2014, 5622 (23.8%) in 2015, 5909 (25.0%) in 2015, and 5552 (23.5%) in 2017, were inspected and included in the final analysis (Fig. 1, Supplementary Fig. 1A, B, C, D, E). The places where the AED was installed were residential settings (49%), multi-use facilities (12%), and schools (10.2%) (Table 1).

**Table 1:** Locations of AEDs.

Facility type	Total (N = 23,619)	2013 (n = 3134)	2014 (n = 3402)	2015 (n = 5622)	2016 (n = 5909)	2017 (n = 5552)
Residential settings	11,564 (48.96)	1778 (56.73)	1882 (55.32)	2738 (48.70)	2640 (44.68)	2526 (45.50)
Multi-use facilities	2732 (11.57)	33,810.78)	280 (8.23)	644 (11.45)	674 (11.41)	796 (14.34)
Schools	2413 (10.22)	74 (2.36)	300 (8.82)	556 (9.89)	1320 (22.34)	163 (2.94)
Public buildings	2126 (9.00)	491 (15.66)	113 (3.32)	759 (13.5)	274 (4.63)	489 (8.81)
Police offices	1515 (6.41)	28 (0.89)	212 (6.23)	294 (5.23)	4778.07)	504 (9.08)
Welfare facilities	929 (3.93)	161 (5.14)	258 (7.58)	11 (0.20)	148 (2.50)	351 (6.32)
Transportation facilities	693 (2.93)	0	0	306 (5.44)	11 (0.19)	376 (6.77)
Industrial facilities	637 (2.70)	148 (4.72)	2 (0.06)	134 (2.38)	159 (2.69)	194 (3.49)
Public health/medical clinics	249 (1.05)	67 (2.14)	70 (2.06)	13 (0.23)	63 (1.07)	36 (0.65)
Hotels and conference venues	181 (0.77)	31 (0.99)	40 (1.18)	26 (0.46)	39 (0.66)	45 (0.81)
Religious facilities	137 (0.58)	5 (0.16)	6 (0.18)	82 (1.46)	20 (0.34)	24 (0.43)
Prisons	7 (0.03)	1 (0.03)	3 (0.09)	1 (0.02)	0	2 (0.04)
Other	436 (1.85)	12 (0.38)	236 (6.94)	58 (1.03)	84 (1.42)	46 (0.83)

Data are presented as n and (percentage) from the total number of AEDs per year.

*AED* automated external defibrillator.

Regarding the factors of AED management, the rate of designation of AED managers had increased from 86.7% in 2013 to 98.9% in 2017 (*p* < 0.0001), and the rate of regular internal check of the AEDs had increased from 85.2% in 2013 to 85.6% in 2017 (*p* < 0.0001). The rate of absence of obstacles near the AEDs had increased from 90.2% in 2013 to 99.1% in 2017 (*p* < 0.0001), and the rate of the AED guide sign installations had increased from 34.3% in 2013 to 91.3% in 2017 (*p* < 0.0001) (Table 2). Regarding the factors of maintenance status and 24-h accessibility, the rate of normal operation status had increased from 94.0% in 2013 to 97.6% in 2017 (*p* < 0.0001), and the rate of good battery status had increased from 95.6% in 2013 to 96.8% in 2017 (*p* = 0.0016). The rate of availability of electrodes had increased from 97.1% in 2013 to 99.0% in 2017 (*p* < 0.0001). However, the rate of validity of electrodes had decreased from 90.0% in 2013 to 87.2% in 2017 (*p* < 0.0001). The rate of 24-h accessibility of the AEDs had decreased from 66.0% in 2013 to 63.4% in 2017 (*p* < 0.0001). The rate of actual use of AEDs ranged from 0.7% to 1.2%, which had not significantly changed during study period (*p* = 0.491) (Table 3).

**Table 2:** AED management status.

Parameter	Total (N = 23,619)	2013 (n = 3134)	2014 (n = 3402)	2015 (n = 5622)	2016 (n = 5909)	2017 (n = 5552)	*p* value
Designation of AED manager	22,434 (94.98)	2717 (86.7)	2985 (87.7)	5551 (98.7)	5688 (96.3)	5493 (98.9)	< 0.0001
CPR and AED training for the manager	21,744 (92.06)	2808 (89.6)	2994 (88.0)	5405 (96.1)	5637 (95.4)	4900 (88.3)	0.2258
A regular check of AED by the manager	20,079 (85.01)	2670 (85.2)	2690 (79.1)	4824 (85.8)	5145 (87.1)	4750 (85.6)	< 0.0001
Absence of obstacles near AED	22,532 (95.39)	2826 (90.2)	3117 (91.6)	5223 (92.9)	5862 (99.2)	5504 (99.1)	< 0.0001
Presence of the AED guide sign	13,937 (59.00)	1074 (34.3)	1092 (32.1)	1996 (35.5)	4709 (79.7)	5066 (91.3)	< 0.0001

Data are presented as n and (percentage) from the total number of AEDs per year.

*CPR* cardiopulmonary resuscitation, *AED* automated external defibrillator.

**Table 3:** Maintenance status, accessibility, and actual use of AEDs.

Parameter	Total (N = 23,619)	2013 (n = 3134)	2014 (n = 3402)	2015 (n = 5622)	2016 (n = 5909)	2017 (n = 5552)	*p* value
Normal operating status	22,893 (96.92)	2947 (94.0)	3289 (96.7)	5463 (97.2)	5776 (97.8)	5418 (97.6)	< 0.0001
Good battery status	22,861 (96.79)	2996 (95.6)	3282 (96.5)	5463 (97.2)	5745 (97.2)	5375 (96.8)	0.0016
Availability of electrodes	23,342 (98.82)	3043 (97.1)	3351 (98.5)	5582 (99.3)	5868 (99.3)	5498 (99.0)	< 0.0001
Valid electrodes	20,578 (87.12)	2822 (90.0)	3117 (91.6)	5113 (91.0)	4685 (79.3)	4841 (87.2)	< 0.0001^†^
24-h accessibility	15,771 (66.77)	2070 (66.0)	2500 (73.5)	3842 (67.5)	3840 (65.0)	3519 (63.4)	< 0.0001^†^
Actual use of the AED	224 (0.99)	22 (0.7)	38 (1.1)	67 (1.2)	56 (0.9)	41 (0.7)	0.4911^†^

Data are presented as n and (percentage) from the total number of AEDs per year.

*AED* automated external defibrillator.

*p* for trend test. ^†^Negative slope indicated a decreasing linear trend.

**Figure 2:**
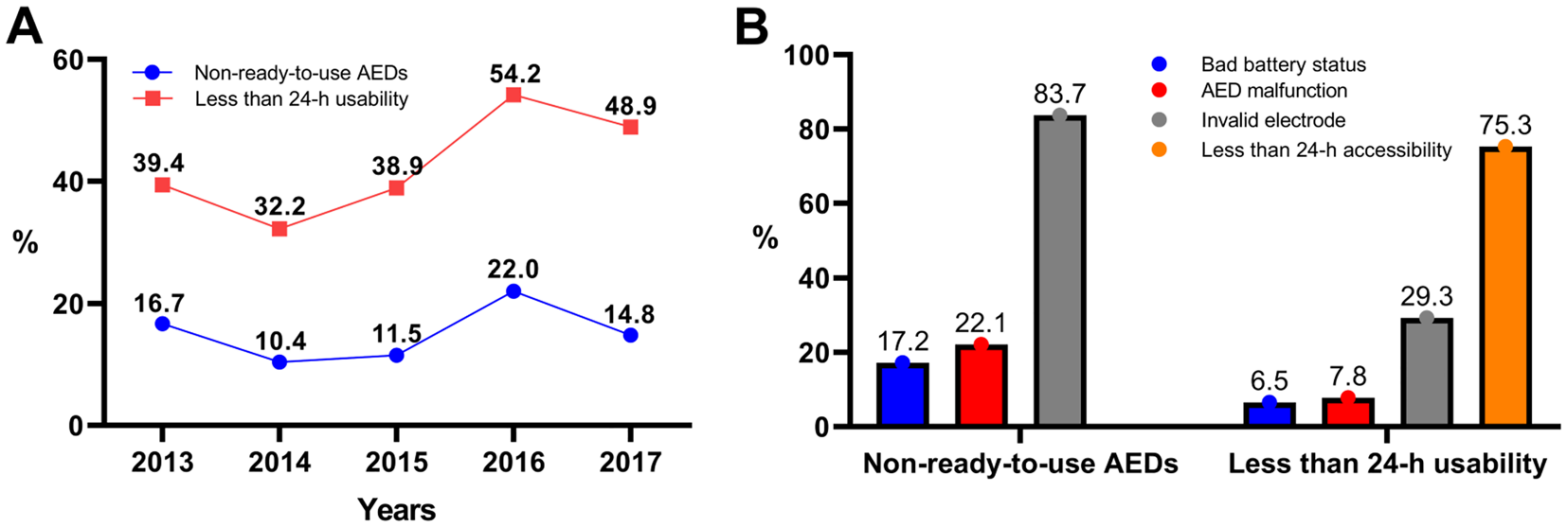
The proportion of non-usable AED and their causes. (**A**) The proportion of non-usable AED. (**B**) Causes of non-usable AED. AED, automated external defibrillator.

In terms of usability, non-ready-to-use AEDs accounted for 15.4% of the total AEDs. The causes of the non-ready-ready-to-use status were invalid electrode (83.7%), AED malfunction (22.1%), and bad battery status (17.2%). AEDs with less than 24-h usability accounted for 44.1% of the total AEDs, and the causes were less than 24-h accessibility (75.3%), invalid electrode (29.3%), AED malfunction (7.8%), and bad battery status (6.5%) (Table 4; Fig. 2). The proportion of the AEDs with less than 24-h usability according to installation location was the highest in residential settings (27.2%), followed by multi-use facilities (18.4%), schools (16.3%), public buildings (16.3), and transportation facilities (Table 5).

**Table 4:** Non-usable AEDs and their causes.

Parameter	Total (N = 23,619)	2013 (n = 3134)	2014 (n = 3402)	2015 (n = 5622)	2016 (n = 5909)	2017 (n = 5552)	*p* value
Non-ready-to-use AEDs^a^	3646 (15.44)	524 (16.72)	354 (10.41)	646 (11.49)	1302 (22.03)	820 (14.77)	< 0.0001
Bad battery status	677 (17.20)	138 (26.34)	113 (31.92)	159 (24.61)	133 (10.22)	134 (16.34)	< 0.0001^†^
AED malfunction	807 (22.13)	187 (35.69)	120 (33.90)	159 (24.61)	164 (12.60)	177 (21.59)	< 0.0001^†^
Invalid electrode	3051 (83.68)	322 (61.45)	285 (80.51)	509 (78.79)	1224 (94.01)	711 (86.71)	< 0.0001
Less than 24-h usability^b^	10,422 (44.13)	1235 (39.41)	1096 (32.22)	2187 (38.90)	3207 (54.27)	2717 (48.94)	< 0.0001
Bad battery status	677 (6.50)	138 (11.17)	113 (10.31)	159 (7.27)	133 (6.08)	134 (4.93)	< 0.0001^†^
AED malfunction	810 (7.77)	187 (15.14)	120 (10.95)	159 (7.27)	164 (7.05)	177 (6.51)	< 0.0001^†^
Invalid electrode	3051 (29.27)	322 (26.07)	285 (26.00)	509 (23.27)	1224 (55.97)	711 (26.17)	< 0.0001
Less than 24-h accessibility	7848 (75.30)	1064 (86.15)	902 (82.30)	1780 (81.39)	2069 (94.60)	2033 (74.83)	< 0.0001

Includes cases with duplicate causes.

^†^Negative slope indicated a decreasing linear trend.

^a^AEDs with bad battery status, malfunction and/or invalid electrode.

^b^AEDs with bad battery status, malfunction, invalid electrode and/or less than 24-h accessibility.

**Table 5:** AEDs with less than 24-h usability according to the installation site.

Facility type	Total (N = 10,439)	2013 (n = 1235)	2014 (n = 1096)	2015 (n = 2187)	2016 (n = 3207)	2017 (n = 2714)
Residential settings	2835 (27.16)	289 (23.40)	255 (23.27)	400 (18.29)	1135 (35.39)	756 (27.86)
Multi-use facilities	1925 (18.44)	283 (22.91)	132 (12.04)	418 (19.11)	451 (14.06)	641 (23.62)
Schools	1704 (16.32)	41 (3.32)	185 (16.88)	411 (18.79)	938 (29.25)	129 (4.75)
Public buildings	1703 (16.31)	401 (32.47)	67 (6.11)	634 (28.99)	232 (7.23)	369 (13.60)
Welfare facilities	705 (6.75)	106 (8.58)	194 (17.70)	5 (0.23)	115 (3.59)	285 (10.50)
Transportation facilities	414 (3.97)	0	0	150 (6.86)	1 (0.03)	263 (9.69)
Police offices	366 (3.51)	13 (1.05)	43 (3.92)	3 (0.14)	161 (5.02)	146 (5.38)
Industrial facilities	207 (1.98)	21 (1.70)	0	66 (3.02)	67 (2.09)	53 (1.95)
Public health/medical clinics	197 (1.89)	49 (3.97)	40 (3.65)	11 (0.50)	63 (1.96)	34 (1.25)
Religious facilities	110 (1.05)	2 (0.16)	5 (0.46)	77 (3.52)	11 (0.34)	15 (0.55)
Hotels and conference venues	35 (0.34)	19 (1.54)	5 (0.46)	4 (0.18)	3 (0.09)	4 (0.15)
Prisons	4 (0.04)	1 (0.08)	1 (0.09)	0	0	2 (0.07)
Other	234 (2.24)	10 (0.81)	169 (15.42)	8 (0.37)	30 (0.94)	17 (0.63)

Data are presented as n and (percentage) from the total number of AEDs per year.

*AED* automated external defibrillator.

## Discussion

On observing public AEDs for 5 years, we found that most AEDs had a relatively good maintenance status, with more than 97% of the AEDs operating normally. However, 15% of the AEDs were not ready for use, and invalid electrode was the most common cause of this. Further, 44% of the AEDs had less than 24-h usability, and less than 24-h accessibility was the most common cause for this. Factors related to management and maintenance of the AED and accessibility to the AED were found to have improved over time. The proportion of valid electrodes decreased over time. Only around 1% of the AEDs were used. This rate did not change during the study period.

For successful implementation of PADs, four essential elements are required, namely planned and practiced response, training of anticipated rescuers in CPR and use of an AED, link to the local emergency medical system (EMS), and a process for continuous quality improvement^11^. In the process of developing and implementing the PAD program, the government or community has paid attention mainly to the installation of AEDs and links to the EMS system through legislation or guidelines^12,13^. In addition, the AED must be maintained in a state of being ready-to-use for 24 h a day. AEDs need to be maintained and tested regularly as per the applicable rules and regulations established by governmental authorities. However, the maintenance and management of AEDs may be the responsibility of the locations holding the AEDs, considering that the community or government may not be able to directly manage the maintenance of the AEDs. Although each country or community has legal provisions for AED registration and management, many public AEDs are not registered in the national registry system or their management status is often unknown. In the Swedish experience, a large proportion (43%) of AEDs was not registered in their registry because of the unawareness of the AED registry or difficulty in registering although those AEDs had high functionality^14^. In a report assessing Canadian public AED registries, governance, and administrative processes across registries were found to be irregular. Some registries do not use a standardized validation or quality surveillance process, which might result in the loss of important information on AED usability, including battery and electrode validity^15^.

In the present study, a trained inspector checked the management and maintenance status of each AED concerning manager, accessibility, equipment, and electrode status by annually visiting the installation site. In addition to the on-site inspection, the inspector trained managers and performed corrective actions against obstacles to the AEDs’ accessibility, such as computers, desks, chairs, banners, etc., which hindered retrieval or visibility of the AED. The maintenance of the defibrillator itself gradually improved during the observation period and obstacles to the AEDs’ accessibility decreased over time. In 2015, the South Korean government revised the AED management regulations, requiring signage to be posted in places where AEDs are installed. As the installation of the signage for AEDs became mandatory, the signage installation rate increased from 35.5% in 2015 to 79.7% in 2016 and 91.3% in 2017. This finding regarding AED signage shows the impact of related regulations on AED management. A significant proportion (44%) of AEDs had less than 24-h usability and this proportion increased over time. In particular, 3 years after the inspection began, the percentage of electrodes that had passed their expiration date was found to have increased. The defibrillator itself does not need a separate function check as it reports data by performing self-tests on its internal circuitry to ensure readiness^16^. Two important accessories, i.e., batteries and electrodes, are subjected to inspection during defibrillator maintenance checks, along with the defibrillator equipment itself. Since the battery is installed in the defibrillator, the charging status can be checked using the indicator. It is checked along with the defibrillator operation status. In contrast, since the electrode is separate from the defibrillator, the validity of the electrode must be checked separately by its expiration date. Therefore, to ensure the working status of the electrode, the manager needs to be aware of the periodic replacement plan. In addition, in cases where the AED is installed with non-governmental external financial support, there is often no financial plan for replacing defibrillator accessories. In such cases, even if the AED manager or inspector finds a problem with the electrodes, the problem cannot be solved. In this respect, when purchasing a defibrillator and installing it in a public space, it is necessary to establish a supply or financial plan for maintaining its accessories along with an inspection plan.

The use of AEDs in public places is related to the number of cardiac arrests in the installation area, the willingness of witnesses to use AEDs, and 24-h usability of the AEDs^17–19^. AEDs are highly accessible during weekdays, but their accessibility declines in the evenings, including nighttime, and on weekends. This limitation in accessibility is associated with the reduced use of AEDs^20^. As observed in our study, the 24-h accessibility was limited for AEDs installed in places that were not open for 24 h, such as multi-use facilities, schools, public buildings, and welfare facilities. In addition, we found that the proportion of less than 24-h accessibility was the highest in residential settings. The limited use of AEDs in a residential setting can be a major hindrance to the PAD program. Only approximately 1% of the AEDs were used. This low utilization rate might be associated with the low 24-h accessibility. Thus, when planning AED installation, it is necessary to consider whether the installation site is open for 24 h. In case the AED is installed in a place that is not open 24 h (e.g. schools), installment of the AED on walls outside the buildings can be considered. Further studies to seek structural and non-structural factors that influence the low AED utilization are needed.

This study has several limitations. The results of this study cannot be generalized to other countries because the AED implementation and maintenance regulations are based on the relevant laws of each country. Because only AEDs funded by Seoul City and not all AEDs in Seoul were included in this study, the spatial density of the AEDs across the catchment area and the spatial gaps in AED coverage were not considered. Additional important factors that affect AED accessibility, such as socioeconomics, rural/urban setting, or EMS stations, were not analyzed. Therefore, this study might lack a holistic context of AED accessibility. There is an EMS-connected alert system using a smartphone application in South Korea. However, the effect of the performance of this system on the accessibility and usability of the AEDs was not accounted for in the study. New AEDs were introduced during the study period, and the environment and maintenance status of the newly introduced AED may have contributed to the improvement of the overall maintenance status. Since the inspectors were recruited annually, the same inspectors did not check defibrillators during the study period. To reduce the bias caused by the inspectors, the recruited inspectors were trained on the inspection method. There is a possibility that their judgment on the study criteria might have changed with time as their inspection process evolved because some inspectors remained the same during the study period.

In conclusion, although the AEDs had a relatively good maintenance status, a significant proportion of public AEDs were not available for 24-h use. Invalid electrodes and less than 24-h accessibility were the main reasons that limited the 24-h usability of public AEDs. Community attention and initiatives are needed to increase the 24-h usability of public AEDs.

## Methods

### Study design and setting

This was a longitudinal, descriptive, observational study using questionnaires and a checklist for AED maintenance. Ethical approval was obtained from the Institutional Review Board of Wonju Christian Hospital, Yonsei University Wonju College of Medicine, Wonju, South Korea (Approval number: CR320033).

The government of South Korea has mandated the installation of AEDs for public housing with more than 500 households, subway stations, passenger terminals, casinos, racecourses, prisons, athletic fields with more than 5,000 seats, and government buildings. These AEDs are required to be registered with the government, and the equipment maintenance status is to be reported annually to the government. Moreover, these AEDs have managers assigned to maintain the equipment and report on equipment status regularly. AEDs installed in places other than the mandatory installation sites must be registered with the government; however, there is no obligation to report their maintenance status or appoint a manager for maintenance.

### AEDs

AEDs installed in Seoul City from 2013 to 2017 were included in this study. Seoul City is the largest metropolitan city in South Korea with an area of 605 km^2^ and a population of about 10 million. Only the AEDs with financial support from the Seoul Metropolitan Government were included in this study irrespective of their installation obligations. AEDs installed with self-funded resources were excluded from this study.

### AED inspection

The Korean Association for Safe Communities (Seoul, Republic of Korea), a non-profit organization registered with the Ministry of Public Administration and Security, South Korea, was commissioned by the Seoul Metropolitan Government to conduct the inspection and surveys of AEDs in Seoul. The AED inspection is conducted by inspectors annually at the sites where the AEDs are installed.

The inspection items for each AED were selected from the checklist recommended by the guidelines for AED placement and management published by the Korean Ministry of Health and Welfare. These selected inspection items were reviewed by an advisory committee composed of two emergency physicians and one specialist each from the AED manufacturing company, AED management company, and consumer protection group. To confirm the suitability of the developed checklist, a pilot inspection of 10 AEDs in different places was carried out. The final version of the inspection checklist included 11 items comprising five items on the AED manager and accessibility, including the designation of the AED manager, CPR and AED training of the manager, regular internal checks, obstacles near the AED, and AED guide sign; four items on the maintenance status, including operation status, battery status, the presence or absence of electrodes, and validity (expiry) of the electrodes; and two items including the 24-h accessibility and actual use of the AED (web-only appendices). The definition of a ready-to-use defibrillator is a defibrillator that has valid electrodes, has a good battery status, and is functioning normally. 24-h accessibility was defined as the AED installation site being open and accessible from outside the building as well as inside the building for 24 h. A 24-h usable defibrillator was defined as a defibrillator that has valid electrodes, has a good battery status, operates normally, and can be used for 24 h. Actual use of the AED was defined as having the AED powered on with the electrodes attached to the patient.

Persons with valid basic life support (BLS) certification were selected as inspectors. A manual that included the concept of PAD, related laws, CPR and AED usage, AED management, survey procedure, survey items, and their definitions, and how to input survey results was developed for training inspectors. The inspectors were trained with a 1-day course (7 h of lectures and 1 h of practice on how to inspect an AED). Through recruitment notices distributed via webforms (http://www.safia.org) and emails, 100 persons with valid certification for BLS were selected and trained to inspect PADs every year from 2013 to 2017.

Every year in November, during the study period, the inspectors visited the AED sites for inspection of the AEDs. The inspector checked each AED using a structured inspection checklist and surveyed the associated manager. After inspecting the AED, the inspector introduced the AED maintenance manual to the manager and allowed the manager to practice how to maintain the AED. Upon completion of the AED inspection, the inspector entered inspection and survey results into a spreadsheet (Excel, Microsoft 2010) and sent it to the data management center, which kept input data and performed quality control.

### Statistical analysis

A retrospective descriptive time series analysis was conducted in this study. Nominal data were calculated as the percentage of the frequency of occurrence and compared using the Chi-square or Fisher’s exact test, as appropriate. The Cochran–Armitage trend test was used to assess temporal trends of the proportion of “yes” responses to the parameters. Ordinary least squares regression analysis was used to estimate linear temporal trends among variables for modeling and analyzing variables. All statistical analyses and spatial distribution analyses. were conducted using R Statistical Software (version 3.6.3; R Foundation for Statistical Computing, Vienna, Austria).

### Meetings

This work has been presented at the European Emergency Medicine Congress 2021.

### Informed consent

This was a retrospective study using AED inspection records. Informed consent was waived by the Institutional Review Board of Wonju Severance Christian Hospital.

### Human rights statement

The study protocol conforms to the ethical guidelines of the 1975 Declaration of Helsinki as reflected in a priori approval by the institution’s human research committee.

## Supplementary Information

Supplementary Figure Legend.

Supplementary Figure 1A.

Supplementary Figure 1B.

Supplementary Figure 1C.

Supplementary Figure 1D.

Supplementary Figure 1E.

Supplementary Information.

## Data Availability

The datasets generated during and/or analysed during the current study are available from the corresponding author on reasonable request.
