# Supplementary material for: Trends in maintenance status and usability of public automated external defibrillators during a 5-year on-site inspection
Source: Sci Rep. 2022 Jun 24;12:10738. doi: 10.1038/s41598-022-14611-1 (PMC9232625; doi:10.1038/s41598-022-14611-1)
Supplement: Supplementary file 1 — Supplementary Figure Legend. [file 41598_2022_14611_MOESM1_ESM.docx]

**Supplementary Figure 1**. Geographical distributions of the AEDs from 2013 to 2017. A. Year 2013, B. Year 2014, C. Year 2015, D. Year 2016, E. Year 2017
