# Supplementary figures and images for: Trends in maintenance status and usability of public automated external defibrillators during a 5-year on-site inspection
Source: Sci Rep. 2022 Jun 24;12:10738. doi: 10.1038/s41598-022-14611-1 (PMC9232625; doi:10.1038/s41598-022-14611-1)

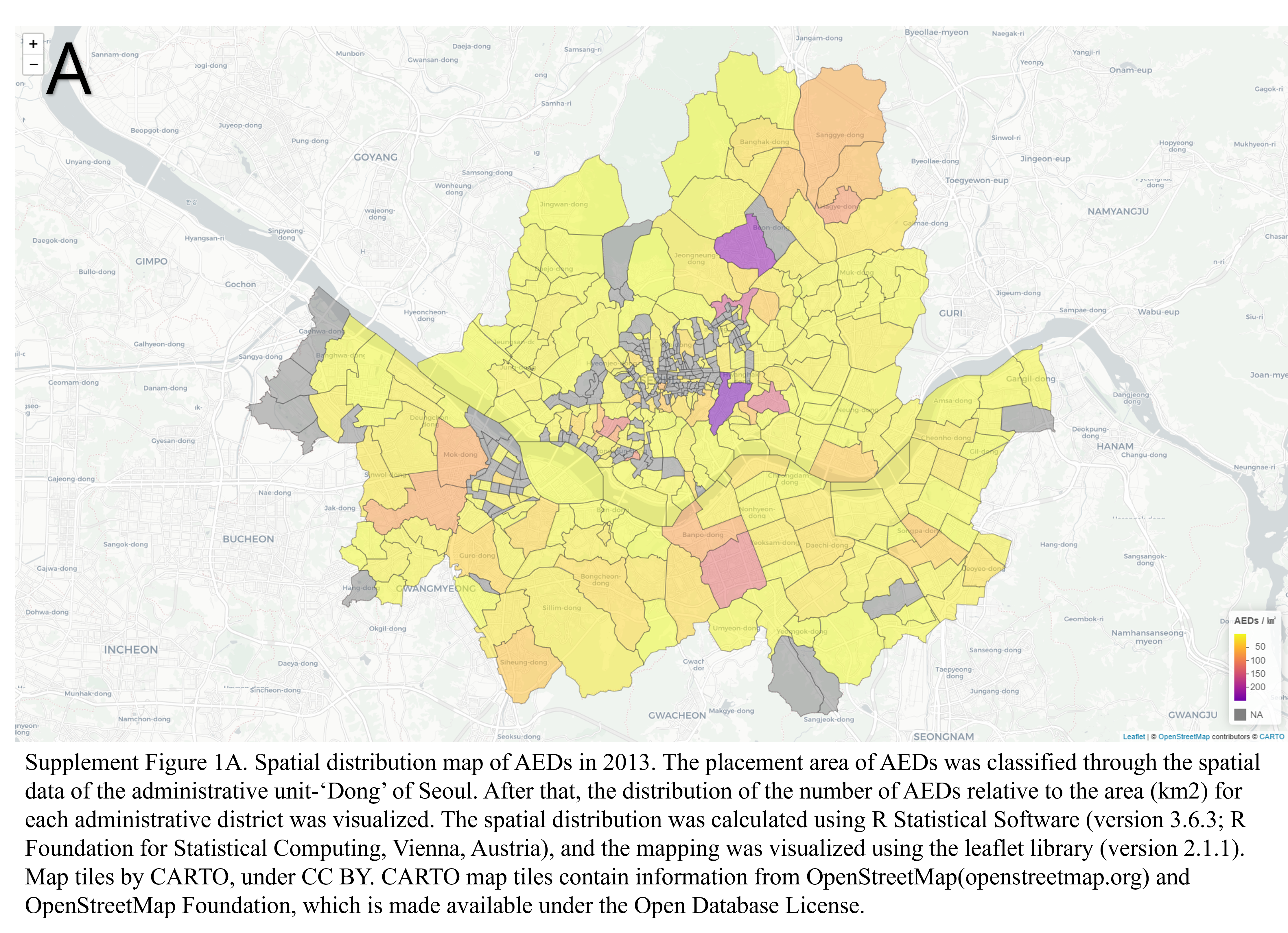

Supplement: Supplementary file 2 — Supplementary Figure 1A. [file 41598_2022_14611_MOESM2_ESM.png]

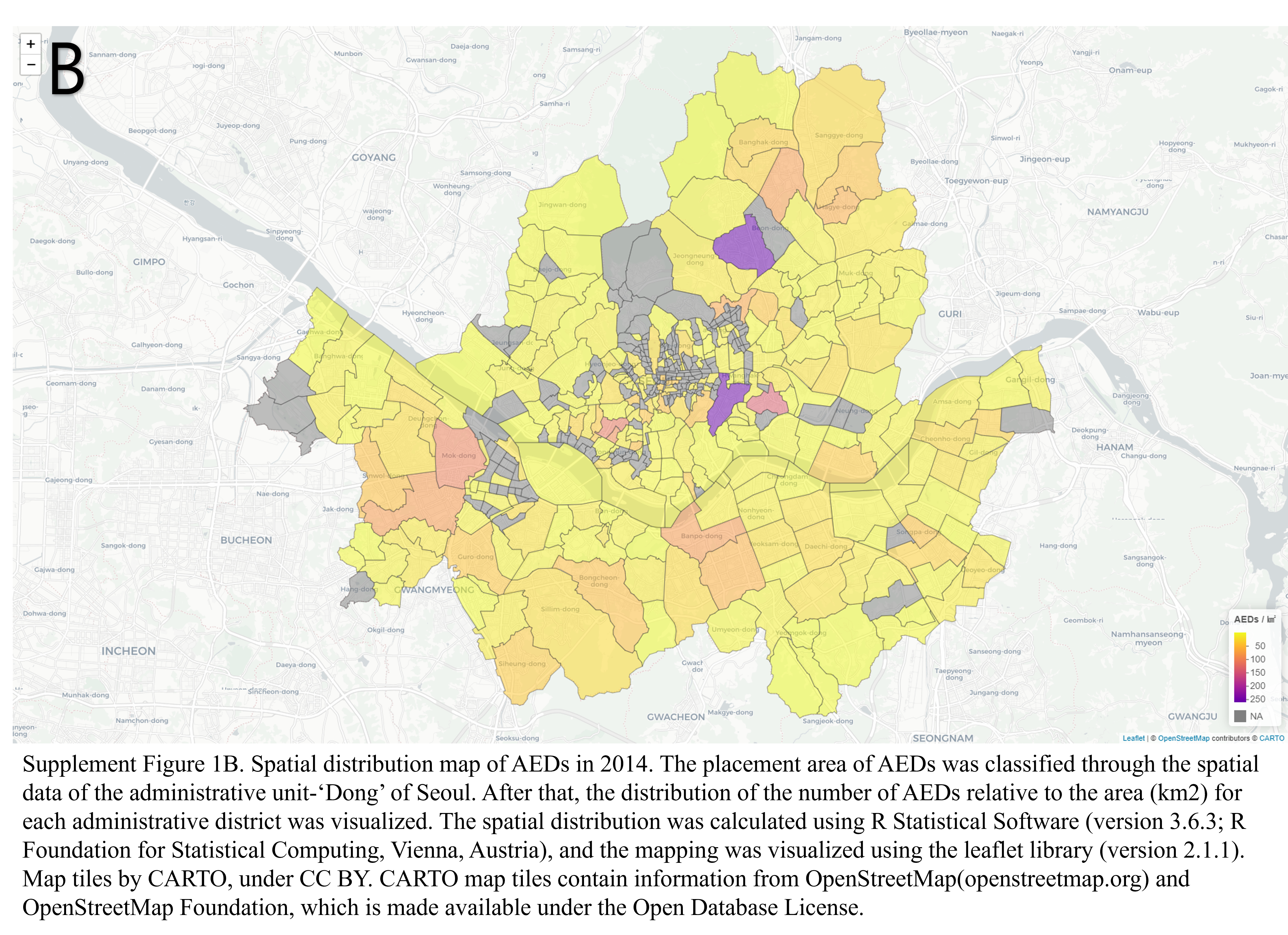

Supplement: Supplementary file 3 — Supplementary Figure 1B. [file 41598_2022_14611_MOESM3_ESM.png]

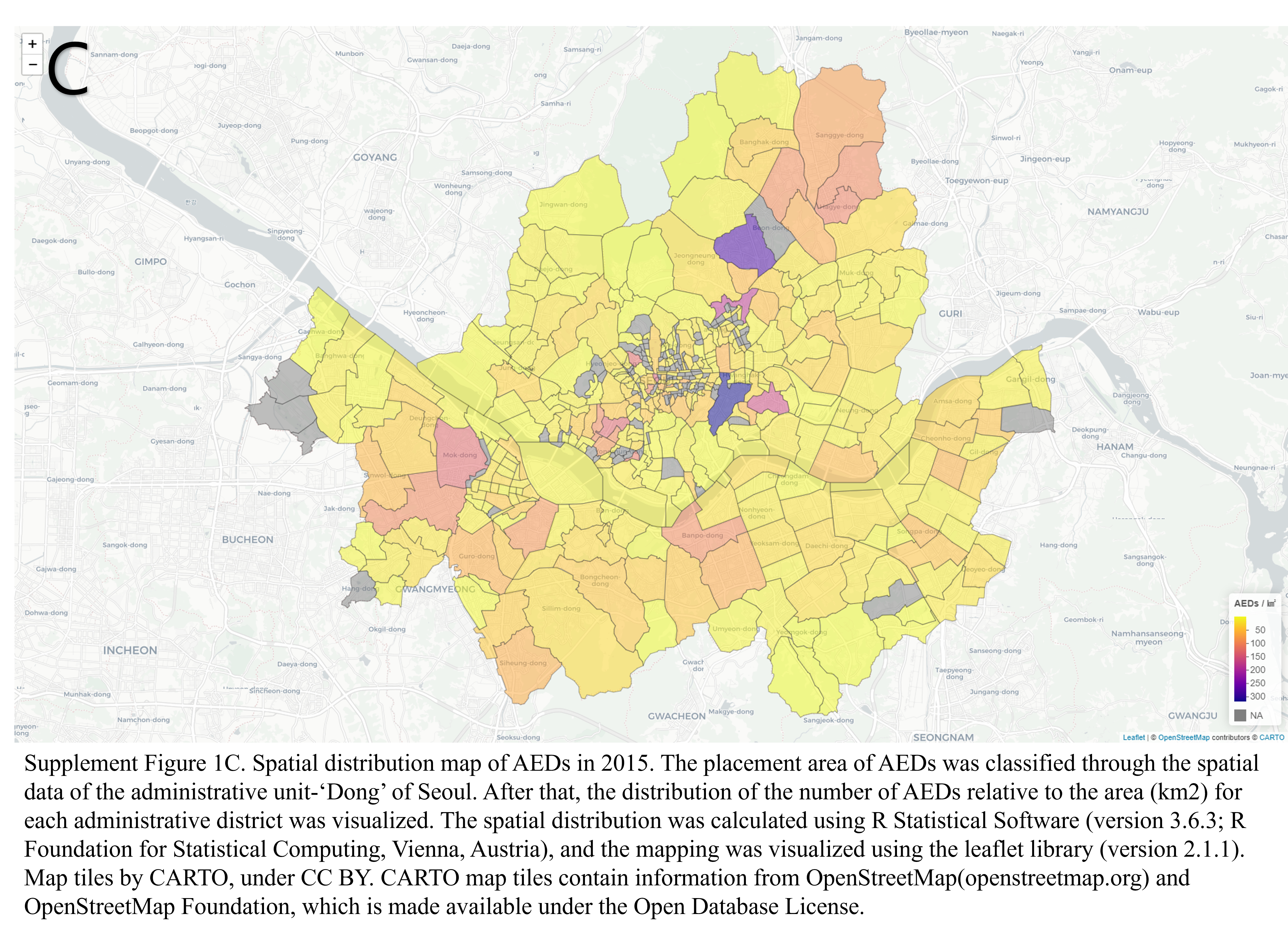

Supplement: Supplementary file 4 — Supplementary Figure 1C. [file 41598_2022_14611_MOESM4_ESM.png]

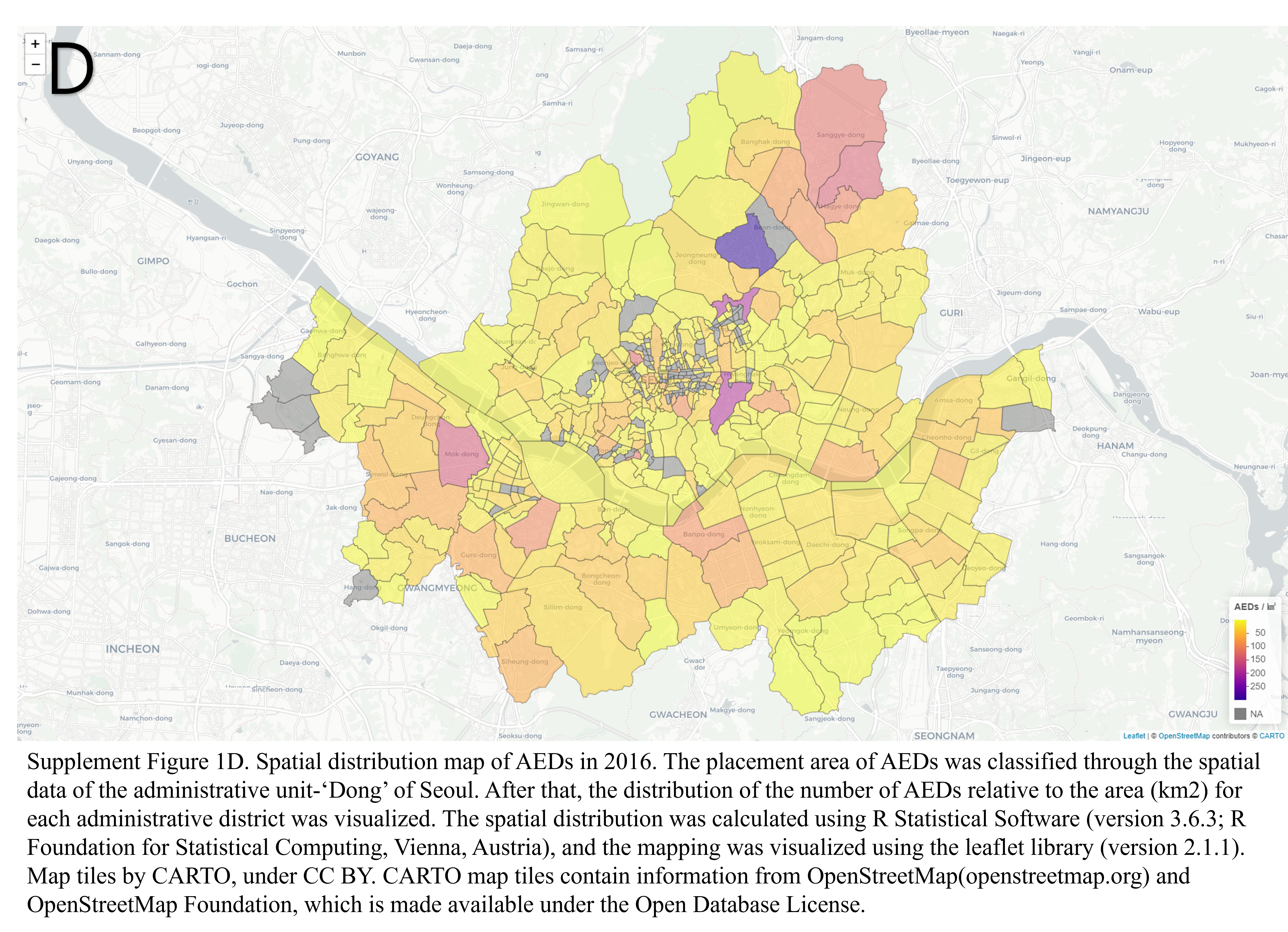

Supplement: Supplementary file 5 — Supplementary Figure 1D. [file 41598_2022_14611_MOESM5_ESM.png]

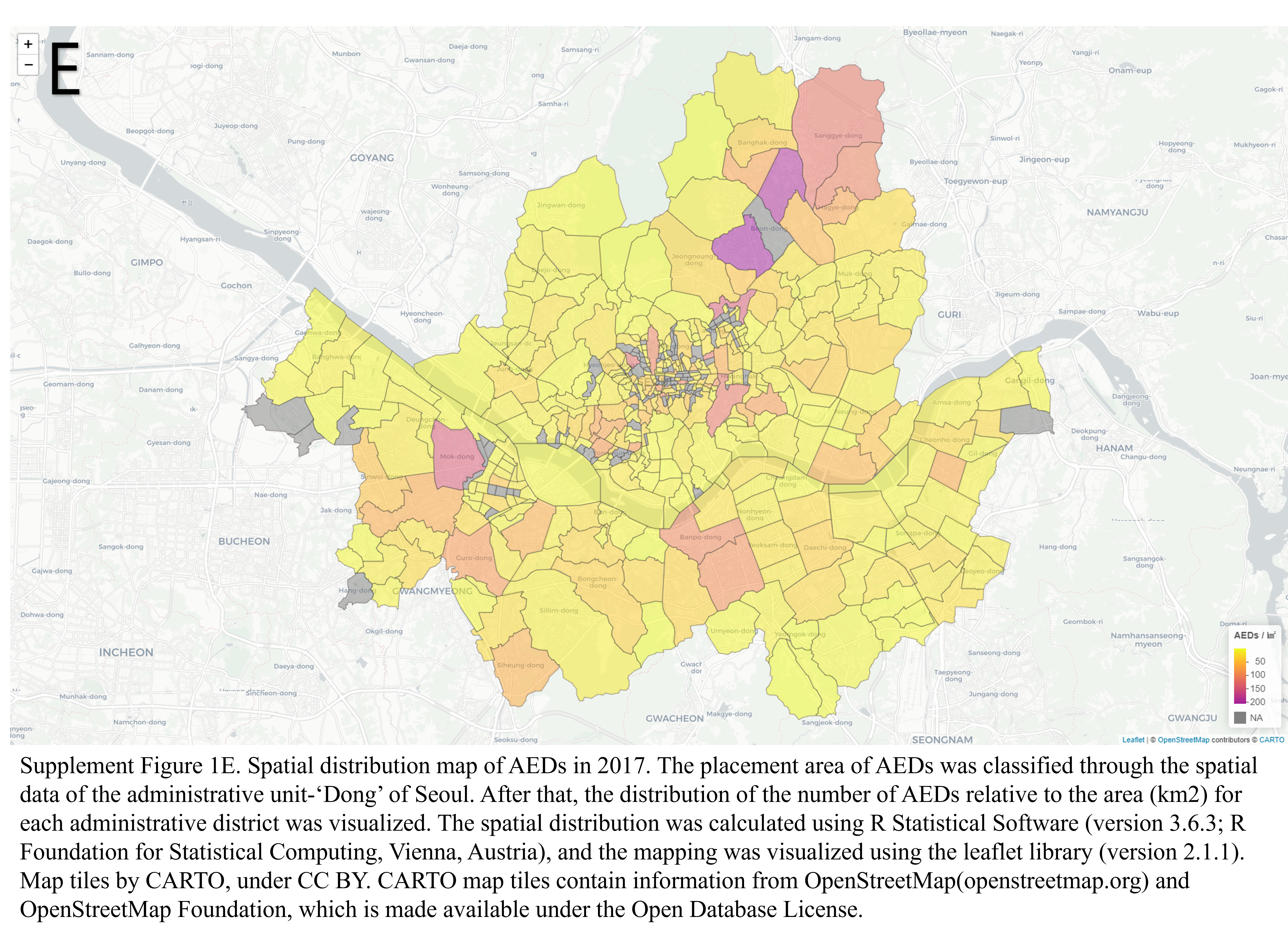

Supplement: Supplementary file 6 — Supplementary Figure 1E. [file 41598_2022_14611_MOESM6_ESM.png]
