# Supplementary material for: Trends in maintenance status and usability of public automated external defibrillators during a 5-year on-site inspection
Source: Sci Rep. 2022 Jun 24;12:10738. doi: 10.1038/s41598-022-14611-1 (PMC9232625; doi:10.1038/s41598-022-14611-1)
Supplement: Supplementary file 7 — Supplementary Information. [file 41598_2022_14611_MOESM7_ESM.docx]

**Web-only appendices**

**Automated external defibrillator check list**

**(For inspector)**

| Institution |  | Address |  |
| --- | --- | --- | --- |
| Installation place |  | | |
| Administrator name |  | Administrator’s contact |  |
| Survey respondent's name |  | Survey respondent's contact | / |
| Date of inspection |  | | |
| Inspector | Name and signature | | |
| Attach photos | ※ **Make sure that the AED installation location and status are visible.**  ⇨ Enter photo and file name: serial number, institution name, location, photo | | |

| **Division** | **Number** | **Check list** | **How to check** | **Results** | |
| --- | --- | --- | --- | --- | --- |
|  |  |  |  | **Y** | **N** |
| **AED**  **Management Status** | **1** | **Is the manager assigned?** | Check whether the manager or assistant manager is designated by interviewing the manager. |  |  |
|  | **2** | **Has the manager completed first aid training?** | Check whether the manager has completed the training on how to use the AED. |  |  |
|  | **2-1** | ***Check the date of completion of training.** | | | |
|  | **3** | **Does the manager know how to use the AED?** | Check whether he has completed the training on how to use the AED. |  |  |
|  | **4** | **Has the manager provided training to employees on performing CPR and using an AED?** | Check whether CPR training and how to use AED were provided to employees. |  |  |
|  | **5** | **Is the manager conducting AED inspections?** | Check whether the manager is using the checklist through interviews and visual inspections. |  |  |
|  | **5-1** | ***Check the inspection method**  **If you are performing an AED inspection,**  **which of the following inspection methods do you use?**  **□ Integrated emergency medical information**  **(http://portal.nemc.or.kr)**  **□ Smartphone app (emergency medical information)**  **□ Checklist (must ask question 5-2)** | | | |
|  | **5-2** | ***Do you check the health center report**  **when using the checklist.**  **Do you report to the public health center**  **when you use the checklist?** | |  |  |
|  |  | ※ AED management and operation  -Operation results of the AED should be reported to the municipality (health center) and the emergency medical information center. | | | |
|  | **6** | **What is the AED inspection cycle?** | | | |
|  |  | ※ The first day of each month is designated as the day of regular maintenance of AED. It is managed to be available at all times by conducting regular maintenance at least once a month.  ※ Article 38-3 of the Enforcement Rules of the Emergency Medical Service Act (Emergency Equipment Management)  ① The founder or manager of a multi-use facility, etc. that has installed the emergency equipment pursuant to Article 47-2 of the Act shall have the person in charge of managing it (1) carry out at least one inspection per month, (2) receive training on emergency equipment use, and (3) be prepared for emergency equipment management. | | | |
| **AED Surrounding Environment** | **7** | **Are there signs of AED device location?** | Check whether the AED device location guide sign is present or not. |  |  |
|  | **7-1** | **How many AED device location signs are there?** | | | |
|  | **8**  *****  **Photographing after field action**  ***** | **Are there any obstacles in the place where the AED is installed?**  **(Check if there is an obstacle that cannot be immediately acted on on-site.)** | Check if there is an object obstructing the AED device.  Clear up obstacles that can be sorted out on the spot (e.g., X-banner, flower pot, etc.). |  |  |
|  |  | **What are the obstacles (impossible to act on immediately) covering the AED device?** ( ) | | | |
|  |  | ***Photographs of field actions and obstacles* ※Photographed to check field actions and obstacles.**  **⇨ Photo file name: Serial number, institution name, obstacle picture** | | | |

| **Division** | **Number** | **Checklist** | **How to check** | **Results** | |
| --- | --- | --- | --- | --- | --- |
|  |  |  |  | **Y** | **N** |
| **AED Type and Operation** | **9** | **Does the voice message appear when the AED power button is turned on?** | Check if the voice message works normally when the AED power button is pressed. |  |  |
|  | **10** | **Is the LED status notification window marked “○”?** | Check if the LED status notification window is displayed as “○” on the front of the AED body. |  |  |
|  | **11** | **Is the battery charged enough?** | Check the charging status of the AED body battery. |  |  |
|  |  | ※ How to check the battery charge status for each device according to manufacturer  ① Mediana: Check if two or more of the three batteries are charged.  ② Philips: Check if “FULL” is indicated.  ③ Nanum Tech: "The battery is insufficient," "Replace the battery." Check if there is no voice guidance or the indicator does not blink.  ④ CU Medical: Check that the status indicator does not appear red. (If it appears red, the battery is low).  ⑤ Nihon Koden: Check if the green “○” is displayed among the ○/× signs.  ⑥ GE Healthcare Korea: Check if the status indicator is green. | | | |
| **AED Pad Status** | **12** | **Are there pads?** | Check whether adult pads are provided in the AED device and the expiration date. |  |  |
|  | **13** | **Are the pads valid for use?** |  |  |  |
|  | **13-1** | **When are the pads valid for use?** | | | |
|  | **14** | ***Only when a pediatric pad is provided**  **Are there pediatric pads?** | Check whether pediatric pads are provided with the AED device and the expiration date. |  |  |
|  | **15** | **Are the pediatric pads valid for use?** |  |  |  |
|  | **15-1** | **When are the pediatric pads valid for use?** | | | |
| **Hourly Availability** | **16** | **When can I use it? Identify the time zones when the AED can be used.** (□ Available 24 h □ Part-time use) | | | |
| **AED Follow-up Management** | **17** | **Have you used AEDs?** | Determine if you have used AEDs in your area. |  |  |
|  | **17-1** | **Did you report to the municipality after use?** | Through interview with the manager, the actual status of management operation after using AED is investigated.  *If you do not report, you will be asked to report to the municipality. |  |  |
|  |  | ※ Article 38-3 of the Enforcement Regulations of the Emergency Medical Service Act (Emergency Equipment Management)  ② If emergency equipment is used, the establishment or manager of the multi-use facility or the person who directly uses it is referred to the Emergency Medical Information Center as per Article 27 of the Act. The Center should informed without delay so that appropriate measures can be taken. | | | |
|  | **17-2** | **Has the AED device been inspected since last use?** | In order to use the AED device in the future, identify the state of the AED device and whether the pads have been replaced. |  |  |
|  | **17-3** | **Are the pads replaced after use?** |  |  |  |
